# Impact of Internet Use on Loneliness and Contact with Others Among Older Adults: Cross-Sectional Analysis

**DOI:** 10.2196/jmir.2306

**Published:** 2013-02-28

**Authors:** Shelia R Cotten, William A Anderson, Brandi M McCullough

**Affiliations:** ^1^University of Alabama at BirminghamDepartment of SociologyBirmingham, ALUnited States

**Keywords:** computers, Internet, loneliness, social isolation, social interaction, older adults, assisted living facilities, independent living

## Abstract

**Background:**

Older adults are at increased risk of experiencing loneliness and depression, particularly as they move into different types of care communities. Information and communication technology (ICT) usage may help older adults to maintain contact with social ties. However, prior research is not consistent about whether ICT use increases or decreases isolation and loneliness among older adults.

**Objective:**

The purpose of this study was to examine how Internet use affects perceived social isolation and loneliness of older adults in assisted and independent living communities. We also examined the perceptions of how Internet use affects communication and social interaction.

**Methods:**

One wave of data from an ongoing study of ICT usage among older adults in assisted and independent living communities in Alabama was used. Regression analysis was used to determine the relationship between frequency of going online and isolation and loneliness (n=205) and perceptions of the effects of Internet use on communication and social interaction (n=60).

**Results:**

After controlling for the number of friends and family, physical/emotional social limitations, age, and study arm, a 1-point increase in the frequency of going online was associated with a 0.147-point decrease in loneliness scores (*P*=.005). Going online was not associated with perceived social isolation (*P*=.14). Among the measures of perception of the social effects of the Internet, each 1-point increase in the frequency of going online was associated with an increase in agreement that using the Internet had: (1) made it easier to reach people (*b*=0.508, *P*<.001), (2) contributed to the ability to stay in touch (*b*=0.516, *P*<.001), (3) made it easier to meet new people (*b*=0.297, *P*=.01, (4) increased the quantity of communication with others (*b*=0.306, *P*=.01), (5) made the respondent feel less isolated (*b*=0.491, *P*<.001), (6) helped the respondent feel more connected to friends and family (*b*=0.392, *P*=.001), and (7) increased the quality of communication with others (*b*=0.289, *P*=.01).

**Conclusions:**

Using the Internet may be beneficial for decreasing loneliness and increasing social contact among older adults in assisted and independent living communities.

## Introduction

As individuals age, they often lose contact with their social network members because of retirement, death of friends and family, and people moving away [1], or communication becomes difficult to maintain due to time or distance. This loss of contact is often associated with declines in socioemotional outcomes, such as feelings of social isolation and increased loneliness. Information and communication technology (ICT) use may help improve socioemotional outcomes by helping older adults overcome time and distance to create or maintain social relationships, thereby decreasing social isolation and loneliness. One particular setting in which loneliness and social isolation may become problematic is in assisted and independent living communities (AICs). Residents of AICs often leave behind social ties when they move from private homes into AICs [2]. The purpose of this study is to examine whether one type of ICT use, specifically Internet use, is related to experiences of loneliness and social isolation among people in independent and assisted living.

### Background

Although aging in place (remaining in one’s home and community) is often cited as the living option preferred by most older adults [3], this is often not a viable option. As people age, they often find themselves in situations where they require more monitoring or care than they can receive living in their home due to declining health or other factors, precipitating a move to an AIC [4]. This type of move often puts older adults at increased risk of feelings of loneliness and social isolation.

Loneliness and social isolation are closely related, yet distinct, concepts. Loneliness is the subjective experience [5] of negative feelings about levels of social contact [6]; otherwise stated, it is the involuntary state of social isolation or the feeling of being alone [7]. Loneliness does not stem solely from objective levels of contact, but rather results from the differences between the levels of desire for social relationships and the availability of relationships [8]. Researchers using loneliness measures typically ask respondents whether they feel lonely, whether they see enough of people, and whether they wish for more contact [6].

Social isolation is the objective experience [5] of the absence of contact with other people [9], especially the absence of contact with people who provide needed or desired social support [6]. Therefore, social isolation is the absence of meaningful social relationships [10]. Although social isolation and loneliness are closely related concepts, the socially isolated person may not report feelings of loneliness even though they lack social contact [1,11]. Conversely, the person who is not socially isolated and has abundant social contact may report feelings of loneliness if that contact is not perceived as fulfilling what the person wants from the relationship [11].

Older adults, in particular, often experience higher rates of loneliness [12,13] and social isolation [1]. This occurs for a variety of reasons, including death of social ties, relocation to different types of living and care communities, and limitations in physical and mental health. In addition, age is negatively related to network size, closeness to network members, and number of primary group ties [14]. Social isolation is a particular problem for older African Americans [15], childless individuals, and widows [6]. Those at risk for loneliness include older adults who have recently migrated following retirement, those caring for a dependent spouse [6], the chronically ill [1], those living alone [7], females, and those living in rural communities [13]. Another risk factor for loneliness among older adults appears to be living in an assisted living facility [2]. A meta-analysis on the influences of loneliness in older adulthood confirmed some of the aforementioned risk factors, such as moving to an institution, having less contact with others, and being female [16]. Another meta-analysis on loneliness in older adulthood showed that decreased levels of physical health, occupying a lower socioeconomic status, and residing in a nursing facility were also risk factors for loneliness among this population [17]. Loneliness does not increase simply because of additional years, but because of an increase in disability and a decrease in social integration [12]. Both loneliness [18] and social isolation [9] are multidimensional concepts, which indicate the need for researchers to examine the social and contextual factors behind the presence or absence of the 2 experiences.

Each of these risk factors for social isolation and loneliness are particularly prevalent among older adults who move to different types of care communities. Older adults who move into assisted living communities are likely to experience loneliness [2]. The importance of familial relationships for such residents combined with dissatisfaction regarding the levels of contact with family members can result in a reduced quality of life for assisted living residents [2]. One possible way to counteract these effects is through Internet use to help maintain social contact with social network ties [19].

### Internet Use, Contact with Others, and Loneliness Among Older Adults

Internet use enables older adults to stay in contact with others [20,21] and communicate with their social ties [22,23]. For example, email is more effective than in-person or phone communication for facilitating regular contact with family and friendship networks [24-29]. A wealth of research indicates that ICT usage may help older adults maintain contact with social ties [20-22,24,27,30-46] with relationships taking place both online and offline [38]. Internet use can also reduce the impact of geographic distance for older adults [45,47], with dispersed families increasingly using the Internet as the primary conduit through which they sustain generational bonds [48].

Older adults lag behind younger age groups in using the Internet. Approximately half of individuals aged 65 and older use the Internet, with 70% of users reporting going online on a usual day [49]. This group is still the least likely to use a computer at home [50]. Social networking site (SNS) use is one Internet application use that has grown exponentially among older adults in the past few years, with just over one-third of Internet users being active on SNSs. They often report doing so to keep in touch with family members. However, email is the primary conduit through which online communication happens for older adult users, with 86% reporting using it. Once older adults are able to cross the digital divide, going online seems to become a usual part of their lives [49]. Unfortunately, older adults aged 75 years and older tend to remain on the wrong side of the digital divide: “Few among this oldest segment of the population are likely to start using the Internet without some assistance and encouragement” [49].

Whether Internet use increases or decreases social isolation is not clear-cut. Although much research has shown Internet use to be of benefit in reducing social isolation and loneliness, other research has found Internet use to be of little or no benefit. Various researchers have found Internet use to be associated with decreases in social isolation and loneliness or to be associated with increases in social connectivity [30,33-39,46-48,51,52]. Use of the Internet has also been shown to enrich the lives of isolated older adults [53], with some older adults reporting lower perceived life stress as a result of ICT use [54]. Likewise, positive associations have been shown between use of the Internet and perceptions of self-efficacy [35].

Other results have not been so positive. Loges and Jung [55] found no relationship between Internet connectedness and social isolation in older adults. Another study of the general population (not just older adults), demonstrated that Internet use had a relatively limited impact on social relationships [56], with still other research indicating that ICT use was associated with an initial decline in social network size and increased loneliness [57]. However, a follow-up study with this same sample done in 2002 showed that Internet users experienced positive effects on communication, social involvement, and well-being [58].

Even when Internet use helps create or maintain relationships, the effects may not fully replicate what has been lost. Nimrod [21], for example, found that relationships constructed in online senior communities are more superficial than offline or real relationships. Results are also likely to vary as a function of the type, amount, timing, and function of Internet usage [59]. If individuals use the Internet for noncommunicative purposes or they are using it in excessive amounts to the detriment of their social roles, it is likely that there will be little impact on their loneliness and social isolation, or that loneliness and social isolation will increase. However, regular usage and use for communicative purposes, such as keeping in touch with social ties and garnering social support, are likely to have positive benefits for older adults. Recent research has shown that going online twice per week was associated with lower levels of loneliness and depression for older adults [60]. As Cotten and colleagues [59] have shown, researchers must go beyond merely including simple measures of Internet usage; they must also examine the type, amount, timing, and function of use because these can influence outcomes in a variety of ways. Although there is much evidence to indicate that Internet use can be beneficial for older adults in overcoming social isolation or loneliness, more research is needed, especially among older adults in continuing care communities.

The purpose of this study was to examine whether frequency of Internet use among older adults in AICs is associated with perceptions of (1) loneliness, (2) perceived social isolation, and (3) the usefulness of the Internet in affecting quantity and quality of communication with social network ties.

## Methods

### Recruitment

The data for this analysis came from an ongoing randomized controlled trial intervention. Alabama, the state where the intervention was conducted, ranked among the lowest in regards to individuals living in households with Internet access [61].

In this study, older adults living in AICs were randomized into 3 groups: (1) ICT (treatment), (2) attention control (placebo), or (3) true control (no treatment or placebo). Older adults living in AICs in the treatment arm were given 8 weeks of training in using computers and the Internet to communicate with family and friends (primarily through email and Facebook) and to find information. Participants in the attention control arm were involved in 8 weeks of activities unrelated to ICTs. Participants in the true control arm did not participate in any intervention activities. Participants from all 3 arms were surveyed 5 times over the course of 1 year: before the 8 weeks (at baseline); at the end of the 8-week intervention; and at 3, 6, and 12 months after the end of the 8-week intervention. Because the purpose of this paper is to examine the relationship among Internet use and outcomes such as loneliness, perceived social isolation, and perceptions of the usefulness of the Internet for staying in touch, ICT users (participants with Internet access) from all 3 arms are included. Additionally, because data collection is not yet complete for all waves of the study, this analysis only uses time 1 (or pretest) data for a cross-sectional analysis. Baseline time 1 data were collected within 1 to 2 weeks of the beginning of any intervention activities. There were 205 participants in the entire sample, with data from 205 participants for the socioemotional analyses, and data from 60 participants for the Internet outcomes because people who responded that they never went online (n=145) were not asked the Internet outcome questions.

### Measures

Our socioemotional outcomes include loneliness, perceived social isolation, and the quality and quantity of communication with others as a result of Internet use. Loneliness was measured with a 3-item version of the UCLA Loneliness Scale [62]. Items in the scale (alpha=.74) were:

1. How often do you feel that you lack companionship?

2. How often do you feel left out?

3. How often do you feel isolated from others?

Responses were measured on a 3-point scale: 1 (hardly ever), 2 (some of the time), and 3 (often). Scores on the individual items were summed to produce the scale.

To measure perceived social isolation, a scale was used (alpha=.69) in which participants were asked how much of the time they were bothered by (1) not having a close companion, (2) not having enough friends, and (3) not seeing enough of the people you feel close to. The responses were coded as 1 (never), 2 (a little of the time), 3 (some of the time), 4 (most of the time), or 5 (all the time). The mean of the 3 scores was used as the scale measure.

Participants who reported going online at least once every few months were asked a series of 7 questions regarding their perceptions of how Internet use had affected their social interactions with others. Participants were asked to what extent they agreed or disagreed with the following statements: “Using the Internet has...” (1) made it easier for me to reach people, (2) contributed to my ability to stay in touch with people I know, (3) made it easier to meet new people, (4) increased the quantity of my communication with others, (5) made me feel less isolated, (6) helped me feel more connected to friends and family, and (7) increased the quality of my communication with others. The responses were coded as 1 (strongly disagree), 2 (disagree), 3 (neither agree nor disagree), 4 (agree), or 5 (strongly agree). These items were assessed individually to better analyze the respondent’s perceptions of the usefulness of the Internet in each specific domain (eg, quality of communication versus quantity; ability to maintain relationships versus establishing new ones).

Internet use was measured simply as frequency of going online. Participants were asked how often they went online: 0 (never), 1 (once every few months), 2 (about once a month), 3 (several times a month), 4 (about once a week), or 5 (several times a week). Only participants who reported having Internet access were included in the analysis because those reporting no Internet access were not asked about their perceptions of how Internet use has affected their communications with others.

### Statistical Analysis

A series of ordinary least squares (OLS) regression analyses were conducted using communications, social isolation, and loneliness as the primary outcomes, and Internet use as the primary independent variable. Analyses controlled for age, the number of social network members (friends and family to whom the participant felt close), study arm (ICT intervention group, attention control group, or true control group), assisted or independent living status, and physical or emotional limitations that would limit social interaction, ie, how much of the time in the past month the participant experienced mental or physical health problems that limited social interaction, measured as 0 (none of the time), 1 (a little of the time), 2 (some of the time), 3 (most of the time), or 4 (all the time). Although we would normally have controlled for race/ethnicity and gender, these controls were not included because most of the sample was white and female.

## Results

### Sample Demographics

As noted, our sample (N=205) was predominantly white (n=194, 94.6%) and female (n=169, 82.4%), with a mean age of 82.8 years (full sample characteristics are presented in Table 1). The sample contained 79 participants who enrolled for ICT training and 126 who had not. On average, study participants had 11.2 friends or family to whom they felt close and appeared unencumbered by physical or mental health issues that might affect their social interaction. The sample was almost evenly split between assisted and independent living residents.

The mean frequency of going online was 1.30 (between once every few months and about once a month), whereas the median frequency of going online was 0.0 with an interquartile range (IQR) of 3.5. Median loneliness was 4.0 (IQR 2.0), indicating low to moderate levels of loneliness in the sample as a whole. Mean perceived social isolation was 1.96, with a median of 1.67, indicating little perception of social isolation. With the exception of “the Internet has made it easier to meet new people,” median scores on the Internet outcome measures were all 4.0, indicating that the sample tended to agree that the Internet had affected their social interactions (summaries of key measures are presented in Table 2).

**Table 1:** Sample characteristics (N=205).

Study variables		Participants
**Sex, n (%)**		
	Male	36 (17.6)
	Female	169 (82.4)
Age, mean (SD)		82.8 (7.7)
**Race/ethnicity, n (%)**		
	White	194 (94.6)
	Other	11 (5.4)
**Study arm, n (%)**		
	ICT intervention	79 (38.5)
	Attention control	72 (35.1)
	True control	54 (26.3)
**Living status, n (%)**		
	In independent living	103 (50.2)
	In assisted living	102 (49.8)

**Table 2:** Summary of key measures (N=205).

Key variables		Mean (SD)	Median (IQR)^a^
**Key independent variable**			
	Frequency of going online	1.30 (2.1)	0.0 (3.5)
Number of close family/friends		11.16 (7.29)	10.0 (8.5)
Physical/emotional limitation to social interaction		0.73 (0.99)	0.0 (1.0)
**Outcomes**			
	Loneliness	4.24 (1.57)	4.0 (2.0)
	Social isolation	1.96 (0.82)	1.7 (1.0)
**The Internet has: (n=60)**			
	Made it easier to reach people	3.73 (1.10)	4.0 (1.0)
	Contributed to my ability to stay in touch	3.87 (1.08)	4.0 (1.0)
	Made it easier to meet new people	2.72 (0.98)	2.5 (1.0)
	Increased the quantity of my communication	3.53 (1.03)	4.0 (2.0)
	Made me feel less isolated	3.60 (0.98)	4.0 (1.0)
	Helped me feel more connected to friends/family	3.73 (1.02)	4.0 (1.0)
	Increased the quality of my communication	3.60 (0.96)	4.0 (1.0)

^a^ IQR: interquartile range

The primary independent variable (frequency of going online) was weakly and negatively correlated with loneliness (Pearson *r*=–0.232, *P*=.001) and social isolation (*r*=–0.134, *P*=.06). Frequency of going online was moderately correlated with the Internet outcome variables, with Pearson correlation coefficients ranging from 0.304 (*P*=.02) (using the Internet has increased the quality of my communication with others) to 0.514 (*P*<.001) (using the Internet has made me feel less isolated). Full correlation results are presented in Tables 3 and 4.

**Table 3:** Correlations (Pearson *r*) among independent variables and outcomes.

Variable	Loneliness (n=205)		Social isolation (n=205)	
	*r*	*P*	*r*	*P*
Frequency of going online	–0.232	.001	–0.134	.06
Number of close friends/family	–0.136	.05	–0.144	.04
Physical/emotional limitations	0.162	.02	0.273	<.001
Age	–0.099	.16	–0.064	.36
In ICT intervention arm	–0.025	.72	–0.065	.35
In attention control arm	0.136	.05	0.170	.02
In assisted living	0.210	.003	0.116	.10

**Table 4:** Correlations (Pearson *r*) among independent variables and answers to the question “Using the Internet has...” (n=60).

Key variables	Using the Internet has...^a^													
	A		B		C		D		E		F		G	
	*r*	*P*	*r*	*P*	*r*	*P*	*r*	*P*	*r*	*P*	*r*	*P*	*r*	*P*
Frequency of going online	0.477	<.001	0.494	<.001	0.314	.01	0.308	.02	0.514	<.001	0.411	.001	0.304	.02
Number of close friends/family	0.065	.62	0.089	.50	0.191	.14	0.144	.27	0.061	.64	0.215	.10	0.186	.15
Physical/emotional limitations	0.126	.34	0.128	.33	0.013	.92	0.227	.08	0.108	.41	–0.048	.72	0.164	.21
Age	–0.088	.50	–0.056	.67	–0.052	.69	–0.081	.54	–0.154	.24	0.111	.40	–0.114	.39
In ICT intervention arm	0.027	.84	–0.034	.79	0.187	.15	–0.126	.34	0.064	.63	0.166	.21	0.065	.62
In attention control arm	–0.163	.21	–0.101	.44	–0.252	.05	–0.204	.12	0.054	.68	–0.287	.03	–0.182	.16
In assisted living	0.018	.89	–0.060	.65	0.031	.81	–0.039	.77	–0.122	.35	–0.017	.90	0.070	.60

^a^ A: made it easier to reach people; B: contributed to my ability to stay in touch; C: made it easier to reach new people; D: increased the quantity of my communication with others; E: made me feel less isolated; F: helped me feel more connected to friends and family; and G: increased the quality of my communication with others.

### Frequency of Going Online and Outcomes

Results of OLS regression analyses showed a relationship between the frequency of going online and socioemotional outcomes (see Table 5) and between frequency of going online and selected Internet-usefulness outcomes (see Table 6). Among the socioemotional outcomes, a 1-point increase in the frequency of going online was associated with a 0.172-point decrease in loneliness scores (*P*=.001) (full results presented in Table 5). After controlling for the number of friends and family, physical/emotional social limitations, age, and study arm, the association remained with a 1-point increase in the frequency of going online being associated with a 0.147-point decrease in loneliness scores (*P*=.005).

**Table 5:** Ordinary least squares (OLS) regressions^a,b^ of socioemotional outcomes (N=205).

Independent variables	Loneliness (score range: 3-9)				Social isolation (score range: 1-5)			
	Model 1		Model 2		Model 1		Model 2	
	*b*	*P*	*b*	*P*	*b*	*P*	*b*	*P*
Constant	4.463	<.001	6.537	<.001	2.028	<.001	2.451	<.001
Frequency of going online	–0.172	.001	–0.147	.005	–0.051	.06	–0.040	.14
Number of family/friends			–0.027	.06			–0.014	.06
Physical/emotional social limitation			0.178	.10			0.200	<.001
Age			–0.028	.05			–.007	.37
In ICT intervention arm			0.123	.65			0.027	.85
In attention control arm			0.304	.27			0.223	.12
In assisted living			0.408	.07			0.058	.61
*F* statistic (df)^c^	11.55 (1, 203)	.001	4.34 (7, 197)	<.001	3.69 (1, 203)	.06	4.17 (7, 197)	<.001
Adjusted *R* ^*2*^	0.05		0.13		0.01		0.10	

^a^ Unstandardized coefficients presented.

^b^ Model 1 uses the key independent variable only. Model 2 adds control variables.

^c^ Degrees of freedom.

Likewise, going online more often was associated with a decrease in the perception of social isolation. A 1-point increase in online frequency was associated with a 0.051-point decrease in respondents’ perceived social isolation (*P*=.06). This relationship, however, failed to hold up in the presence of the controls with a 1-point increase in frequency of going online being associated with a statistically nonsignificant 0.040-point decrease in perceived social isolation (*P*=.14).

Among the measures of perception of the social effects of the Internet (see Tables 6-9), all outcomes showed a statistically significant relationship with frequency of going online. Each 1-point increase in the frequency of going online was associated with a 0.508-point increase in agreement that using the Internet had made it easier to reach people (*P*<.001); a 0.516-point increase in agreement that using the Internet had contributed to the respondents’ ability to stay in touch (*P*<.001); a 0.297-point increase in agreement that using the Internet had made it easier to meet new people (*P*=.01); a 0.306-point increase in agreement that using the Internet had increased the quantity of respondents’ communication with others (*P*=.01); a 0.491-point increase in agreement that using the Internet had made the respondent feel less isolated (*P*<.001); a 0.392-point increase in agreement that using the Internet helped the respondent feel more connected to friends and family (*P*=.001); and a 0.289-point increase in agreement that using the Internet had increased the quality of respondents’ communication with others (*P*=.01).

There were no consistent patterns of association between the control variables and the outcomes.

**Table 6:** Ordinary least squares (OLS) regressions^a, b^ for ⁘using the Internet has made it easier to reach people and contributed to my ability to stay in touch⁙ (n=60).

Independent variables	Made it easier to reach people				Contributed to ability to stay in touch			
	Model 1		Model 2		Model 1		Model 2	
	*b*	*P*	*b*	*P*	*b*	*P*	*b*	*P*
Constant	1.661	.002	3.240	.03	1.763	.001	2.492	.09
Frequency of going online	0.467	<.001	0.508	<.001	0.475	<.001	0.516	<.001
Number of family/friends			0.021	.35			0.023	.31
Physical/emotional social limitation			0.015	.91			0.039	.77
Age			–0.024	.18			–0.013	.45
In ICT intervention arm			–0.196	.54			–0.300	.34
In attention control arm			–0.370	.28			–0.214	.52
In assisted living			0.288	.35			0.074	.81
*F* statistic (df)^c^	17.094 (1, 58)	<.001	3.136 (7, 52)	.01	18.737 (1, 58)	<.001	3.175 (7, 52)	.007
Adjusted *R* ^*2*^	0.21		0.20		0.23		0.21	

^a^ Unstandardized coefficients presented.

^b^ Model 1 uses the key independent variable only. Model 2 adds control variables.

^c^ Degrees of freedom.

**Table 7:** Ordinary least squares (OLS) regressions^a,b^ for ⁘using the Internet has made it easier to meet new people and increased the quantity of my communication with others⁙ (n=60).

Independent variables	Made it easier to meet new people				Increased quantity of communication with others			
	Model 1		Model 2		Model 1		Model 2	
	*b*	*P*	*b*	*P*	*b*	*P*	*b*	*P*
Constant	1.508	.003	2.690	.06	2.280	<.001	2.673	.07
Frequency of going online	0.273	.01	0.297	.01	0.283	.02	0.306	.01
Number of family/friends			0.040	.06			0.024	.28
Physical/emotional social limitation			–0.052	.69			0.170	.21
Age			–0.022	.17			–0.007	.70
In ICT intervention arm			0.249	.40			–0.603	.06
In attention control arm			–0.333	.29			–0.632	.06
In assisted living			0.322	.26			0.149	.62
*F* statistic (df)^c^	6.358 (1, 58)	.01	2.237 (7, 52)	.05	6.086 (1, 58)	.02	2.526 (7, 52)	.03
Adjusted *R* ^*2*^	0.08		0.13		0.08		0.15	

^a^ Unstandardized coefficients presented.

^b^ Model 1 uses the key independent variable only. Model 2 adds control variables.

^c^ Degrees of freedom.

**Table 8:** Ordinary least squares (OLS) regressions^a,b^ for ⁘using the Internet has made me feel less isolated and helped me feel more connected to friends and family⁙ (n=60).

Independent variables	Made me feel less isolated				Helped me feel more connected to friends and family			
	Model 1		Model 2		Model 1		Model 2	
	*b*	*P*	*b*	*P*	*b*	*P*	*b*	*P*
Constant	1.619	.001	3.211	.01	2.076	<.001	1.685	.23
Frequency of going online	0.447	<.001	0.491	<.001	0.374	.001	0.392	.001
Number of family/friends			0.022	.25			0.042	.05
Physical/emotional social limitation			0.009	.94			–0.091	.48
Age			–0.027	.07			–0.001	.96
In ICT intervention arm			0.266	.33			0.035	.91
In attention control arm			0.383	.18			–0.444	.16
In assisted living			–0.096	.71			0.189	.51
*F* statistic (df)^c^	20.876 (1, 58)	<.001	4.171 (7, 52)	.001	11.806 (1, 58)	.001	3.090 (7, 52)	.008
Adjusted *R* ^*2*^	0.25		0.27		0.16		0.20	

^a^ Unstandardized coefficients presented.

^b^ Model 1 uses the key independent variable only. Model 2 adds control variables.

^c^ Degrees of freedom.

**Table 9:** Ordinary least squares (OLS) regressions^a,b^ for ⁘using the Internet has increased the quality of my communication⁙ (n=60).

Independent variables	Increased the quality of my communication			
	Model 1		Model 2	
	*b*	*P*	*b*	*P*
Constant	2.449	<.001	3.482	.01
Frequency of going online	0.260	.02	0.289	.01
Number of family/friends			0.042	.05
Physical/emotional social limitation			0.094	.46
Age			–0.022	.17
In ICT intervention arm			0.031	.92
In attention control arm			–0.269	.39
In assisted living			0.427	.13
*F* statistic (df)^c^	5.917 (1, 58)	.02	2.213 (7, 52)	.05
Adjusted *R* ^*2*^	0.08		0.13	

^a^ Unstandardized coefficients presented.

^b^ Model 1 uses the key independent variable only. Model 2 adds control variables.

^c^ Degrees of freedom.

## Discussion

### Key Results

Our findings indicate that Internet use was associated with lower levels of loneliness among residents of AICs. Given recent research showing that loneliness among the older adult population is associated with a higher chance of fulfilling the criteria for metabolic syndrome [63] and an increased risk of death [64], the maintenance of personal relationships through the Internet could be critical to well-being for this segment of the population. Moreover, among the general population, using the Internet to maintain communication with family and friends has been associated with well-being [65], further providing support for the idea that going online could be beneficial for older adults.

Our results, however, suggest that the frequency of going online impacts loneliness, but not perceptions of social isolation, with higher frequency associated with lower levels of loneliness but not with lower levels of perceived social isolation. It may be that perceptions of social isolation are related more to face-to-face contact than online contact with network ties; thus, frequency of going online is not related to perceived isolation. Unfortunately, our data do not allow us to further explore this relationship. Although mixed, these results support prior research showing that Internet use positively impacts quality of life among older adults [19-21,33-37,39,44-45,53,66,67].

Perhaps unsurprising among a group of self-motivated Internet users, participants tended to agree that using the Internet had a positive effect on their social relationships, making it easier to reach people, stay in touch, meet new people, feel less isolated, and feel more connected to friends and family. It is interesting that frequency of going online was not associated with our social isolation scale; however, frequency of going online was associated with participants agreeing that using the Internet made them feel less isolated. Although they may perceive that the Internet is useful in this particular way, simply measuring frequency of going online is not sufficient to impact social isolation. Participants also tended to agree that using the Internet had increased both the quantity and quality of their communication with others. Unfortunately, our measure does not allow us to speak to the degree of this change, only to the degree of agreement that each one has increased.

Of note is that the strength of these various relationships varies greatly. For example, the relationship between frequency of going online and agreement that the Internet had made it easier to reach people, contributed to my ability to stay in touch, made me feel less isolated, and helped me feel more connected to friends and family were all comparatively strong, with coefficients ranging from 0.392 to 0.516. Much weaker were the associations between frequency of going online and agreement that the Internet had made it easier to meet new people, increased the quantity of communication with others, and increased the quality of my communication with others, with coefficients ranging from 0.289 to 0.306. Taken together, these results suggest the perception that the Internet is comparatively better at facilitating established communications, even perhaps replacing older communications methods. The Internet is comparatively worse at affecting either the quantity or quality of communications or helping to establish new relationships.

Although other studies have found that older adults report the quality of social contact being more important than the quantity of social contact [68], our results suggest that the more important contrast is between the ability of the Internet to help simply maintain relationships and the ability (or lack thereof) of the Internet to help deepen relationships or create new ones, at least among older adults. This may be related to the previous finding that online relationships may be perceived as more superficial than other relationships [21]. Thus, in terms of using the Internet to help alleviate the effects of loneliness or social isolation, it may be enough to use the Internet to simply stay in touch or feel like one is a part of what is going on in the world, as opposed to attempting to use the Internet to create and/or maintain deep, personal relationships.

Although recent data have shown communication with others to be a primary reason why older adults go online [49], some might still be reluctant to adopt the Internet as a way of connecting with others, thereby placing them at further risk of loneliness and social isolation. Regardless of older adults’ level of motivation and reasons for going online, ICT training and interventions could enable them to cross the digital divide [22,68-70] and employ ICTs as a way to alleviate loneliness.

### Limitations

Limitations of the current study include the small sample size, the lack of diversity in terms of gender and race/ethnicity, and lack of measures of disability, caregiving, migration, chronic health conditions and pre-AIC levels of social integration, and that the study was only conducted in AICs in Alabama. Another limitation of the present study is that it did not measure participants’ expectations about how going online might impact levels of loneliness and social isolation. As reported elsewhere, computer acceptance is motivated by older adults’ expectations of how computer usage will help them achieve what they deem valuable [71]. An important variable to include in analyses such as the ones presented here, for example, could be whether participants were going online with the intention of connecting with others. Similarly, additional measures assessing the type, timing, amount, and function of Internet use could provide further insights into these relationships [59]. Also, further research is needed on how technology usage may impact older adults not living in AICs and how these processes may vary as a function of gender, race/ethnicity, severity of health impairment, and region of the country. Given that only cross-sectional data were used, the results of this study indicate associations between key measures but should not be seen as reflective of causal relationships.

### Conclusions

In sum, this research contributes to the work in this area by showing that Internet usage has positive benefits for older adults living in AICs. Given that this population experiences high rates of loneliness and depression, with psychosocial resources providing a buffer for depression [72] and personal social networks enhancing well-being [73], encouraging older adults to begin using the Internet to communicate with others could help to enhance social contact and decrease loneliness. As formal care homes are able to encourage social engagement between residents [74], continuing existing ICT programs and beginning new ones in communities without programs could be beneficial for fostering relationships among residents as well as with others in their social networks.

## Abbreviations

AIC: assisted and independent living community
ICT: information and communication technology
IQR: interquartile range
OLS: ordinary least squares
SNS: social networking site
